# Multiomic Landscape Uncovers TRMT112 as a Central Driver of HPV-Positive Head and Neck Squamous Cell Carcinoma

**DOI:** 10.1155/humu/5308441

**Published:** 2025-11-04

**Authors:** Tongnan Yin, Qian Guo, Zhenwei Wen, Yikun Guo, Chenwen Li, Zhongyu Qu

**Affiliations:** ^1^Central Laboratory, Nanyang Central Hospital, Nanyang, China; ^2^Department of Rhinology, The First Affiliated Hospital of Zhengzhou University, Zhengzhou, China; ^3^Department of Stomatology, Nanyang Central Hospital, Nanyang, China; ^4^Department of Pulmonary, Beijing University of Chinese Medicine Shenzhen Hospital (Longgang), Shenzhen, China; ^5^Department of Dermatology, Henan Provincial People's Hospital, Henan University People's Hospital, Zhengzhou, China; ^6^Department of Oncology, Nanyang Central Hospital, Nanyang, China

## Abstract

Head and neck squamous cell carcinoma (HNSCC) ranks second among men and sixth globally, with a notable increase in HPV-associated cases. However, the molecular underpinnings and immune landscape of HPV+ HNSCC remain incompletely understood. In this study, we first retrieved and harmonized single-cell RNA sequencing (RNA-Seq), bulk RNA-Seq, and spatial transcriptomic profiles from public repositories. We then applied high-dimensional weighted gene coexpression network analysis (hdWGCNA) and gene nonnegative matrix factorization (GeneNMF) to dissect HPV+ epithelial subpopulations, their extracellular matrix (ECM)–interacting ligand programs, and CXCL/complement immune circuits. Furthermore, we mapped the spatial niches of malignant and immune cells and constructed a consensus prognostic index using 101 machine learning algorithms. Our findings revealed transcriptionally distinct HPV+ epithelial clusters that activate viral oncogenesis, inflammatory pathways, and ECM-sensing pathways. These cells communicate with stromal and immune compartments via CXCL axes and complement cascades, yet they are spatially segregated from lymphocytes. A high-risk signature, identified as HPV-related risk genes including TRMT112, stratified the TCGA-HNSC and GSE65858 cohorts into patients with markedly worse 1-, 2-, and 3-year survival rates (ROC–AUC 0.934, 0.968, and 0.973) and poor responses to immunotherapy. Notably, TRMT112 expression inversely correlated with cytotoxic T-cell infiltration, mechanistically linking it to the formation of “cold” tumors. Our integrative analysis defines HPV-driven epithelial subpopulations whose TRMT112-enriched, immune-excluded microenvironment contributes to therapeutic resistance, thus providing robust prognostic biomarkers and actionable targets for precision immunotherapy in HPV+ HNSCC.

## 1. Introduction

Head and neck tumors are among most common malignant tumors. More than 90% of the tumors originate from malignant lesions of the squamous epithelial cells of the head and neck area. The oral cavity, pharynx, and larynx are sites of occurrence of head neck tumors. The pharynx includes nasopharynx, oropharynx, and hypopharynx [1, 2]. According to estimates provided by GLOBOCAN, there are around 931,000 new cases of head and neck tumors each year. Furthermore, this also leads to 467,000 deaths each year. This type of tumor is ranked seventh among all other malignant tumors. Additionally, the head and neck tumor patients exhibit a suicide rate of 63.4/100,000, which is the second highest among all malignant tumors worldwide [3]. Recent studies have found that the persistent infection of human papillomavirus (HPV) has become one of the new independent risk factors for head and neck squamous cell carcinoma (HNSCC) [4, 5]. In the various countries, although the incidence shows a difference, there is a constant and significant increase in the incidence of HPV-related HNSCC [6]. Distinct clinical and pathological features characterize HPV-positive HNSCC. The study shows that HPV+ HNSCC is characterized by nonkeratinizing and undifferentiated basaloid squamous cell carcinoma histopathologically [5, 7–9]. HPV install into host cells' genome showing derepression and overexpression of E6 and E7 oncogenes which induce persistent HPV infection. Moreover, it promotes the ubiquitination and degradation of the tumor suppressor protein, p53. On the contrary, E7 protein can suppress the retinoblastoma protein (pRb) expression and initiate DNA synthesis (S phase) in the cell cycle. In addition, HPV infection activates oncogenes while also inducing the suppression of apoptosis and cellular immortalization. As the uncontrolled increase of the HPV and the expression of the viral oncogenes alters the tumor microenvironment (TME). This results in complex communication between HPV ± HNSCC cell.

HPV-positive nonsmall cell carcinoma (NSCC) is clinically, histologically, and molecularly different from HPV-negative NSCC, the researchers reported. HPV-positive NSCC is more sensitive to radiotherapy and chemotherapy and has a significantly better prognosis than HPV-negative NSCC [10, 11]. Numerous research studies in the field have confirmed that the immune response mounted against HPV is beneficial for better outcomes and prognosis of HNSCC. The immune response might be a better prognosis than the actual clinical stage of the tumor [3, 12, 13]. Nonetheless, the exact carcinogenic molecular mechanisms and pathways involved in HPV-associated HNSCC are still not well understood to date. The plethora of available bulk sequencing datasets and the limited number of single-cell sequencing of human cancer samples make the integration of bulk and single-cell RNA sequencing a feasible, cost-effective, and clinically translatable option for cancer subtyping, stratification, and prognosis, especially with substantial assistance from robust computational algorithms [9, 13, 14]. The purpose of this study is to identify key HPV+ HNSCC subtypes by performing targeted clustering with scRNA-seq and bulk transcriptomics. Furthermore, the TME interactions and spatial characteristics in spatial transcriptomics will be analyzed, including patient survival. In conclusion, the findings provide novel perspectives in biological insights, molecular stratification, and therapeutics in HNSCC.

## 2. Methods

### 2.1. Data Resource

Based on the literature [15], single-cell data were integrated from CZ CELLxGENE, resulting in a total of 63,537 cells. Environmental contamination was removed using decontX. The PercentageFeatureSet function was used to calculate percentages of mitochondrial, ribosomal, and erythrocyte genes in cells that expressed over 200 genes, with a percentage of mitochondrial gene expression under 20% and an erythrocyte gene proportion below 1%. The normalized merged scRNA-seq data was used with the FindVariableFeature function to identify the Top 2000 most variable genes. The ScaleData function was used to scale all genes, and the RunPCA function was used to perform dimensionality reduction on the Top 2000 highly variable genes. Batch correction was conducted using the Harmony algorithm. The FindNeighbors and FindCluster functions were applied to identify cell clusters (resolution = 0.8). Next, we applied the UMAP method to further reduce dimensionality. Lastly, we employed the FindAllMarkers function to annotate our analysis and visualize our results with the references as well as CellMarker 2.0 [15, 16]. We used the inferCNV package to identify tumor cells. Subsequently, tumor cell subpopulations underwent pseudotime analysis using the Monocle2 package. Additionally, the authors performed Ro/e analysis to validate the expression differences among cell subpopulations (an enrichment greater than 1.5 indicates high enrichment, while less than 0.5 indicates sparsity of the subpopulation) [17]. Spatial transcriptomics uses spatial transcriptomic data from references to identify samples in HNSCC. The HPV-negative samples HN481 and HN487 and the HPV-positive samples HN482 and HN483 were selected [15]. In addition, transcriptomic data for HNSCC and pancancer with survival information were obtained from the University of California Santa Cruz (UCSC) database (https://xenabrowser.net/), and the GSE65858 dataset was downloaded from the GEO database for subsequent transcriptomic level validation [18]. All data required for the analysis of this study can be obtained from publicly available sources. The limma package was used to get the DEGs between the tumor and normal using the filter, *p* value < 0.05 [19].

### 2.2. GeneNMF Deconvolution of Epithelial Subpopulations and Prognostic Marker Extraction

Statistical approaches to high-dimensional data make nonnegative matrix factorization (NMF) a popular method to acquire sparse features from nonnegative data vectors. The method is specifically designed to decompose single-cell sequencing data, thereby simplifying large complex matrices (genes by cells) into a few interpretable gene programs. GeneNMF is a package that implements the NMF method for single-cell omic data analysis [20, 21]. The function can be directly used on a Seurat object to perform dimensional reduction of the data along with detecting robust gene programs across other samples. Using the GeneNMF program, we carried out consistent NMF for malignant epithelial subpopulations across samples to obtain robust and consistent epithelial cell subpopulations for further subsequent analyses. For GeneNMF, we have specified that the parameter *k* ranges from 4 to 9, the metric used is “cosine,” and the number of multiplicative updates (nMP) is set to 6. Next, we performed prognostic analyses on robust subpopulation markers generated from transcriptomics to discover important prognostic subpopulations.

### 2.3. High-Dimensional Weighted Gene Coexpression Network Analysis (hdWGCNA) Identification of HPV+-Associated Coexpression Modules in Malignant Epithelium

hdWGCNA is a software used to carry out WGCNA on high-dimensional transcriptomic data such as single-cell RNA-Seq or spatial transcriptomics [22]. Due to its modules, it can build coexpression networks across multicellular and spatial hierarchies. hdWGCNA finds the most important modules of connected genes as well as the context (biological meaning) of these modules. Using hdWGCNA, the most important HPV+-related modules were identified via the comparison of the distribution of key HPV expression in malignant epithelium.

### 2.4. SCENIC Analysis

SCENIC is utilized to recognize the different states of cells in single-cell data and construct regulatory networks. The pySCENIC analysis based on Python 3.10 investigates the regulatory network of transcription factors (TFs) in malignant tumor subgroups. Using the RcisTarget database, enrichment analysis detects regulatory networks between transcription start sites and genes. Visualization is conducted using the pheatmap package [23].

### 2.5. Functional Enrichment and Metabolic Pathway Activity Analysis

Gene Ontology (GO) analysis is widely used in large-scale functional enrichment analysis, including biological processes, molecular functions, and cellular components. The Kyoto Encyclopedia of Genes and Genomes (KEGG), which compiles information on genomes, biological pathways, diseases, and drugs, is a much-used resource [24]. Using the clusterProfiler R package, we performed GO annotation and KEGG pathway enrichment analyses of the DEGs. A *p* value of less than 0.05 was considered statistically significant [25].

### 2.6. Cell Communication

For cell communication analysis, we have used the R package CellChat (V1.6.0) based on single-cell data and our cell classifications. With CellChatDB.human as a reference, we investigated the interactions between the cells and studied the relationships of 32 pathways among the cells [26]. In this study, we used NicheNet to gather protein–protein interaction data and regulatory factor interaction data from different databases. We specifically picked out endothelial cells as the ligands to clarify the downstream signaling targets, which were verified in CellChat [27].

### 2.7. Spatial Transcriptomics

We conducted our analysis using the Seurat 4.0 package in R, based on the recommended data processing guidelines available at https://satijalab.org/seurat/articles/spatial_vignette.html. The SCTransform function was used for data normalization. Then, PCA and UMAP were performed for dimension reduction and clustering. SpatialFeaturePlot function was used to visualize gene expression features, and SPOTlight was used for deconvolution and visualization. Apart from that, we did cell colocalization analysis with CARD.

### 2.8. Construction and Validation of Prognostic Models

Using the survival data from TCGA, a univariate Cox analysis was performed on the filtered differential genes using bootstrap kernels in order to identify key differential prognostic genes [28]. We incorporated a total of 10 algorithms of machine learning as well as 101 algorithm combinations in order to produce consensus IRLS, which is of high accuracy as well as stable performance. The algorithms include random survival forest (RSF), Elastic Net (Enet), LASSO, Ridge, Stepwise Cox, CoxBoost, Cox partial least squares regression (plsRcox), supervised principal component (SuperPC), generalized boosted regression model (GBM), and survival support vector machine (survivalSVM). The process goes as follows: (a) Key prognostic genes are obtained by bootstrap-based univariate Cox regression analysis, and then, the intersection is taken with the key genes from single-cell malignant subgroups to obtain the critical genes. (b) Then, 101 algorithm combinations are taken on the critical genes to fit predictive models based on the leave-one-out cross-validation (LOOCV) framework in the TCGA-HNSCC cohort. (c) All models are validated in the validation set (GSE65858). (d) For each model, the Harrell's concordance index (C-index) is calculated in all validation datasets, and the model with the highest average C-index is selected as the best model.

### 2.9. Prognostic Analysis and Grouping

Using the survival package and with the 101 process, we computed the optimal cutoff value of HPV-related high-risk genes (HRGs) using MRG scores. Subsequently, we conducted high grouping and low grouping based on this cutoff value and plotted the Kaplan–Meier curves according to the grouping. Next, we used the rms package to analyze and visualize nomogram, calibration, and DCA curves.

### 2.10. Prognostic Model of Immune Infiltration and Prediction of Immunotherapy

We utilized the ESTIMATE algorithm based on expression data to assess the stromal and immune cell scores in malignant tumor tissues [29]. Using the IOBR package, we calculated the immune cell distribution differences between high and low prognosis groups using four methods: CIBERSORT, EPIC, MCPCounter, and TIMER [30–34]. The IMvigor210 data queue is a Phase II clinical trial investigating the use of atezolizumab for the treatment of bladder cancer [35]. We assess the efficacy of immunotherapy based on the queue evaluation model. Additionally, we utilize the submap algorithm to predict the degree of response to immunotherapy in the prognostic model [36].

### 2.11. Screen Potential Therapy Agents for Patients With High GTS

We used gene set enrichment analysis (GSEA) to analyze the activation status of carcinogenic pathways in high-risk patient groups. Expression data for human cancer cell lines (CCLs) were obtained from the Broad Institute Cancer Cell Line Encyclopedia (CCLE) [37]. The CTRP V2.0 (https://portals.broadinstitute.org/ctrp) and PRISM Repurposing datasets (19Q4; https://depmap.org/portal/prism/) were utilized to obtain drug sensitivity data for CCLs. After this, we calculated the area under the curve (AUC) for high and low groups to further assess drug sensitivity.

### 2.12. Cell Culture

This study utilized developmental oral keratinocyte (DOK) and HNSCC (FaDu) cell lines. The cell lines were sourced from the ATCC cell bank and the Chinese Academy of Sciences cell bank. DOK cells were cultured in 1640 medium (Gibco, United States), while the FaDu cell line was maintained in DMEM medium. All culture media were supplemented with 10% fetal bovine serum (FBS; Gibco, United States) and 1% penicillin/streptomycin (C100C5, NCM Biotech, China). The cell lines were incubated in a humidified chamber at 37°C with 5% CO_2_. All experiments were conducted using mycoplasma-free cells.

### 2.13. Quantitative PCR Analysis

Total RNA was extracted using the RNeasy Plus Mini Kit (Qiagen, Hilden, North Rhine-Westphalia, Germany, 74,136). Reverse transcription was performed with 100 ng of total RNA using the PrimeScript RT Reagent Kit (Takara, Kyoto, Shimogyo-ku, Japan, RR037A). The SYBR Green detection system (Applied Biosystems, Carlsbad, California, 4,367,659) and the specific primer TRMT112 (also from Qiagen) were utilized. Normalization was conducted using the housekeeping gene GAPDH, and the mRNA levels in each experimental group were normalized relative to the control group. The primer sequences for the target genes are listed as follows: TRMT112 (forward: TTTGTGCGGCGACATGAAAC, reverse: TTTCGGCACCTGGATCAGAC) and GAPDH (forward: TTCTTTTGCGTCGCCAGCC, reverse: TTCTCAGCCTTGACGGTGCC).

### 2.14. Immunohistochemistry

Formalin-fixed, paraffin-embedded tissue sections (4 *μ*m) were deparaffinized in xylene and rehydrated through graded ethanol. Endogenous peroxidase activity was blocked with 3% hydrogen peroxide (Servicebio, Wuhan, China) for 25 min at room temperature, followed by antigen retrieval in citrate buffer (pH 6.0, Servicebio) using microwave heating for 15 min. Sections were blocked with 3% bovine serum albumin (Servicebio) for 30 min at room temperature and incubated overnight at 4°C with rabbit anti-TRMT112 primary antibody (Proteintech, Cat# 26472-1-AP, 1:250). After washing, slides were treated with HRP-conjugated goat anti-rabbit IgG (Servicebio, Cat# GB23303, 1:200) for 50 min, and staining was visualized with DAB (Servicebio) followed by hematoxylin counterstaining. Stained sections were examined using a Nikon E100 microscope (Nikon Instruments, Tokyo, Japan). Quantitative image analysis was performed with QuPath (Version 0.6.0). Positive cell detection was conducted using hematoxylin OD as the detection channel, with a background radius of 8 *μ*m, a sigma of 1.5 *μ*m, and nuclear area constraints of 10–400 *μ*m^2^. Intensity thresholds for nuclear DAB OD were set at 0.1, 0.2, and 0.4 to classify weak (1+), moderate (2+), and strong (3+) staining. H-score and Allred score were subsequently calculated for statistical evaluation.

## 3. Statistical Analysis

Data analysis and graphical representation were conducted using R Version 4.3.1 and GraphPad Prism 9.0. To compare two groups, either the Wilcoxon test or the *t*-test was employed, while one-way ANOVA was applied for comparisons involving multiple groups. The correlations between variables were evaluated using Pearson correlation coefficient. A significance level of *p* values less than 0.05 was used to indicate statistical significance.

## 4. Results

### 4.1. Single-Cell Landscape

**Figure 1:**
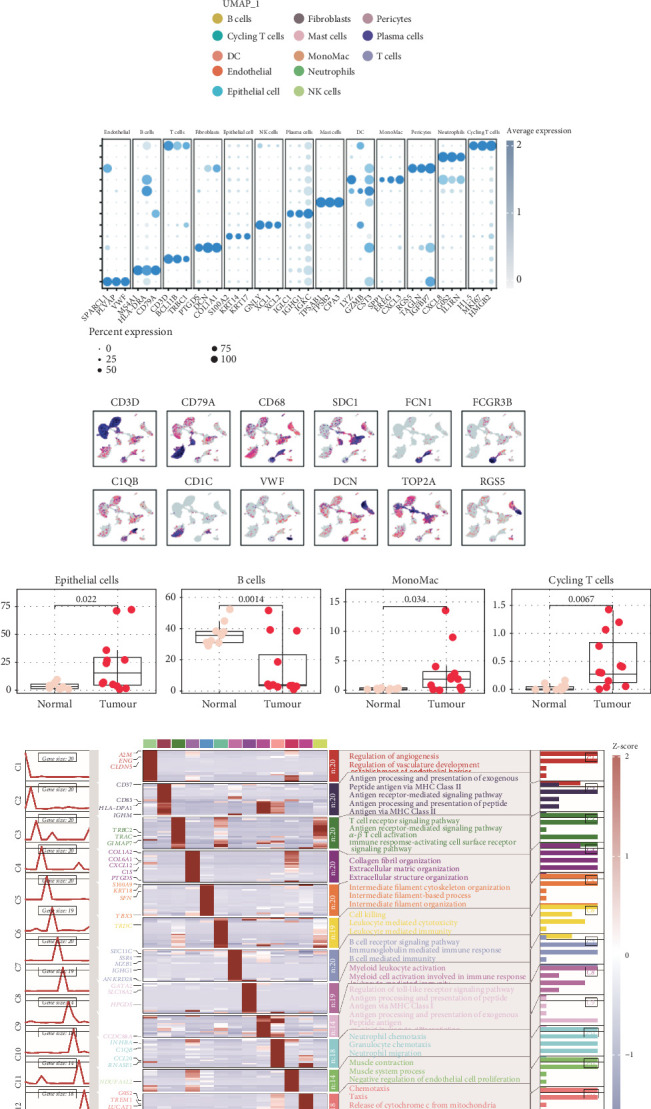
Single-cell landscape. (a). Single-cell UMAP plot. (b) Dot plot of Top 3 markers for cell subpopulations and feature plot of genes located on UMAP. (c) Analysis of the differences in subpopulation ratios between tumor and normal tissues. (d) Annotation of cell subpopulations and enrichment heatmap.

The data was annotated, and we identified 13 cell types in total including T cells, cycling T cells, B cells, MonoMac, endothelial cells, fibroblasts, pericytes, DC, B cells, neutrophils, mast cells, plasma cells, and epithelial cells (Figure 1a). The accuracy of cell annotations and subtype location was further confirmed through the enrichment of marker genes, as well as the Top 3 markers for each subtype (Figure 1b). Subsequent functional enrichment analyses of single-cell subpopulations matched our annotations (Figure 1d), showing the accuracy of our annotations. Moreover, differential analysis of the subpopulations of single cell indicates that the epithelial cells, monomacrophages, and cycling T cells were significantly upregulated in tumor samples, while B cells were significantly downregulated in tumor tissues (Figure 1c).

### 4.2. The hdWGCNA and GeneNMF Reveal Key Epithelial Subpopulations Associated With HPV

**Figure 2:**
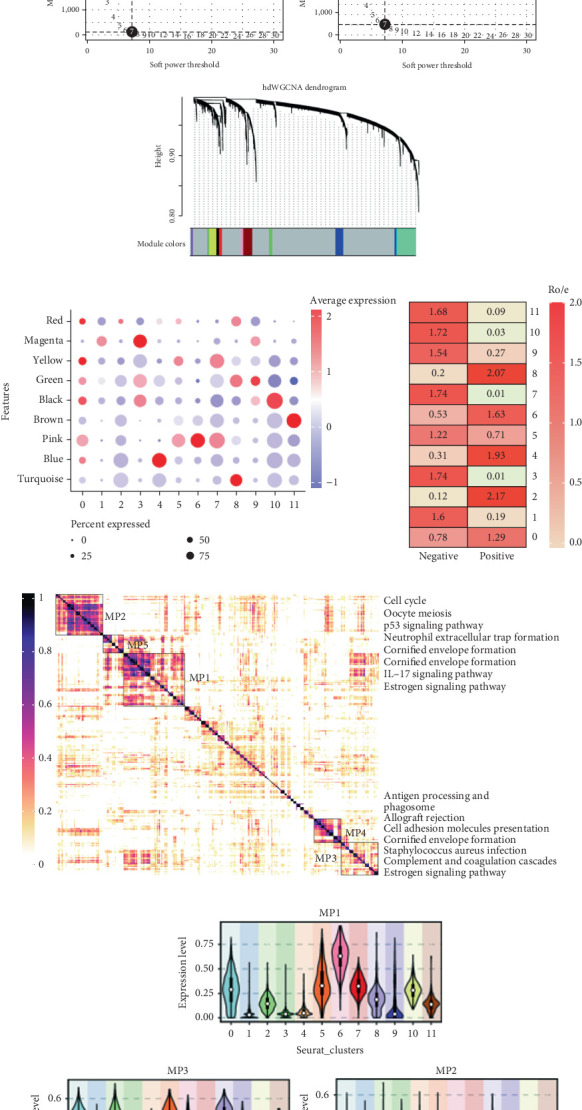
hdWGCNA and GeneNMF reveal key epithelial subpopulations associated with HPV. (a) UMAP visualization of differences between HPV+ and HPV− epithelial subpopulations. (b) Selection of soft power in hdWGCNA. (c) Dendrogram. (d) Dot plot of enrichment of different modules in clusters. (e) Heatmap of enriched modules and functional enrichment from GeneNMF. (f) Violin plot of MP enrichment in clusters. (g) Prognostic survival curves for MP genes.

We visualized the epithelial subpopulation first for the HPV+ and HPV− subgroups. The results showed that subgroups differ significantly (Figure 2a). Another analysis we conducted was Ro/e analysis, which revealed the strong enrichment of HPV+ epithelium in Subgroups 2, 4, 6, and 8 (Figure 2d). For subsequent analysis based on subgroup differences, we conducted hdWGCNA analysis where the soft threshold was determined as 7 (Figure 2b). Subgroup 8 was significantly enriched in turquoise and green (Figure 2b,c). To further elucidate the key subgroups, we introduced GeneNMF to characterize important malignant epithelial modules in HPV-mediated HNSCC. Through clustering, we find five modules (model program, MP). According to the results of the enrichment analysis, we observed that MP1 is mainly enriched in the estrogen signaling pathway and IL-17 signaling pathway and MP2 is mainly enriched in the cell proliferation pathway. The formation of cornified envelopes and the complement and blood coagulation cascades primarily involves MP3. MP4 is related to antigen processing and presentation, phagosome, and allograft rejection pathways, whereas MP5 is significantly enriched in cornified envelope formation (Figure 2e). The enrichment levels of MP1–5 in the subgroups are shown by the violin plot. We show that MP1, MP3, and MP4 show no specific expression in the subgroups. MP2 is specifically enriched in 8. MP6 is specifically enriched in MP5 (Figure 2f). The transcriptomic validation of the MP genes showed that only MP2 displayed a poorer prognosis (Figure 2g). In our prior analyses, we found that Cluster 8 likely represents a pathogenic subgroup of HPV+ epithelium.

### 4.3. Functional Analysis of HPV Epi

**Figure 3:**
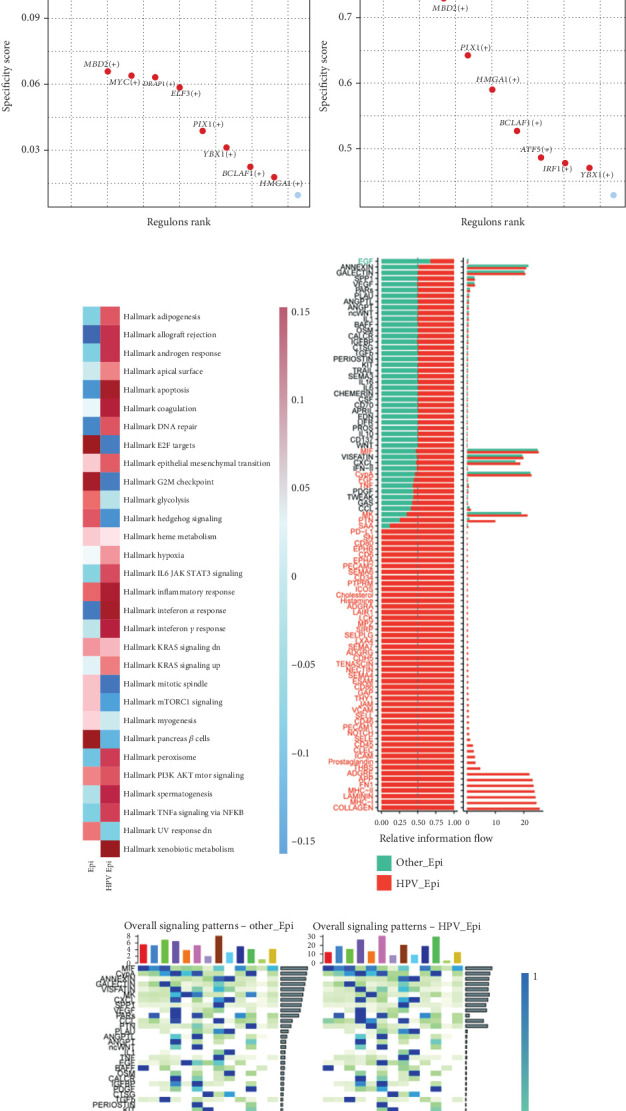
Functional analysis of HPV Epi. (a) Pseudotime analysis of HPV Epi. (b) Volcano plot of HPV Epi and Epi SCENIC Top 10 transcription factors. (c) Differential GSVA analysis of HPV Epi and Epi. (d) Differential pathways of cell communication between HPV Epi and Epi. (e). Heatmap of differential cell communication.

**Figure 4:**
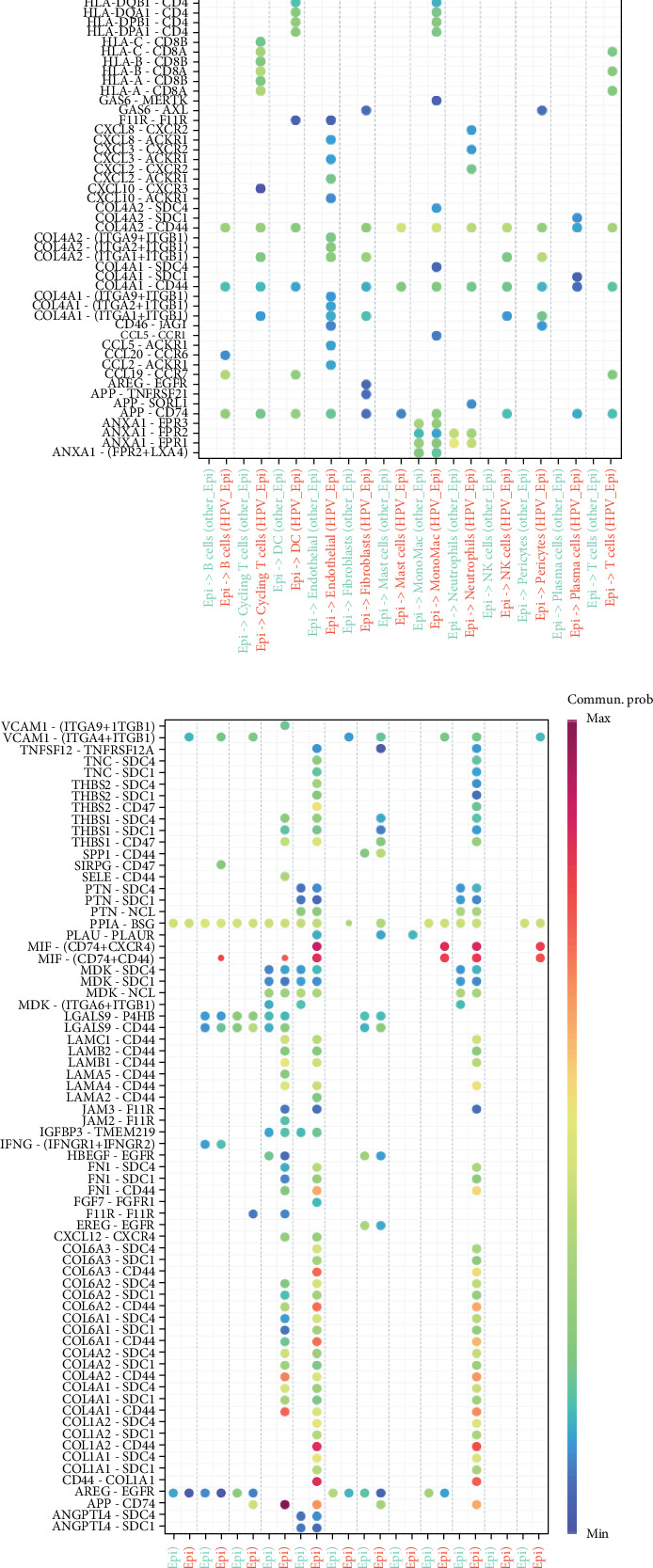
Cell communication point map. (a) Cell communication point map when HPV Epi acts as a ligand in cell communication with Epi. (b) Cell communication point map when HPV Epi acts as a receptor in cell communication with Epi.

We characterized HPV Epi as epithelial cells having a score greater than the MP2 gene expression median and localized to Cluster 8 subgroup. According to pseudotime analysis, HPV epithelial cells are situated at the tail end of the differentiation trajectory of the cells (Figure 3). According to SCENIC analysis, the HPV Epi is distinguished from normal Epi in terms of the expression of several TFs. In particular, IRF1, which mainly regulates interferon-related functions, is significantly enriched along with ATF5, an inflammatory-related TF (Figure 3). The results of GSVA analysis showed that the functions of HPV Epi are associated with inflammation-related pathways such as apoptosis, hypoxia, and inflammatory response, as well as other pathways closely related to viruses including the ones mediated by EMT and INF-*α* (Figure 3). After this, differential cell communication analysis showed that HPV Epi communicated significantly differently from other malignant epithelia. The HPV Epi cells also had a strong expression of immune pathways like MHC-II and PD-L1 (Figure 3). Results of ligand point maps on further cell communication analysis show that when ligands are epithelial cells, significant differences in communication between HPV-positive epithelial cells (HPV Epi) and other epithelial cells (Epi) with fibroblasts, pericytes, and endothelial cells were observed. To be precise, pathways linked to cell perception and response to ECM—mostly its mechanical and biochemical properties—like COL4A1 − (ITGA9 + ITGB1), were primarily enriched for HPV Epi, as were communication pathways that boost proliferation, like VEGFB–VEGFR1. Another major CXCL and complement cascade reaction-related communication between HPV Epi and immune cells, which is absent in other Epi cells, are shown in Figure 4. In addition, when HPV Epi functions as a receptor for cell communication, there are ECM and TGF-*β*-associated communication responses between fibroblasts and HPV Epi, such as THBS2-SDC1 (Figure 4). This tells us that there is a complex dialogue between the experimental cell and stromal cell which could further promote the occurrence of HNSCC.

### 4.4. Construction of Prognostic Models Based on Machine Learning

**Figure 5:**
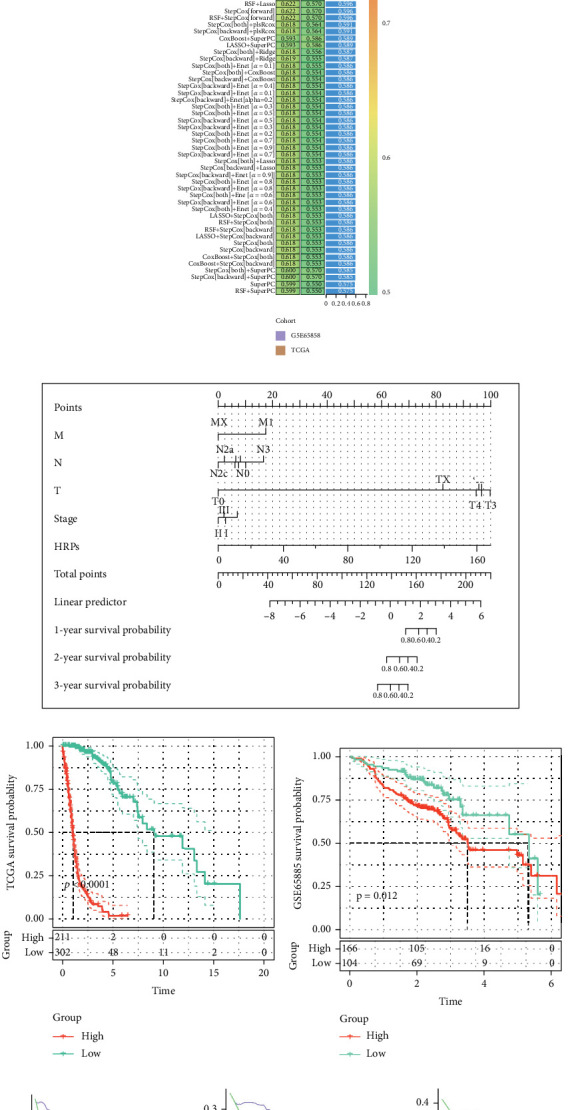
Construction of prognostic models based on machine learning. (a) GSEA analysis of key differential module genes. (b) Univariate Cox forest plot of key differential module genes. (c) Algorithm for constructing the optimal prognostic model in 101. (d) Nomogram. (e) Survival prognostic curve of HRGs from TCGA. (f) Survival prognostic curve of HRGs from GSE65858. (g). One- to three-year DCA curve. (h) Prognostic restricted cubic spline. (i) Time-dependent ROC curve. (j) Multivariate Cox analysis of model genes.

We acquired key differential module genes based on the critical genes of the module and the differential genes obtained from TCGA-HNSC (abs(log2FC) > 0.8 and *p* < 0.001), followed by GSEA analysis. According to the results, the functions of module genes were primarily enriched in key pathways like inflammatory response, TNF-NF*κ*b, INF-*α*, and apoptosis (Figure 5a). Our bootstrap screening of differential prognostic genes led to the selection of 11 genes (Figure 5b). Subsequently, we used 101 machine learning algorithms to find out the best prognostic model based on these 11 differential prognostic genes (Table S1). On the basis of the number of genes and C-index, we recommend StepCox[backward] + RSF as the optimal prognostic model, among which the HRGs were identified as TRMT112, APP, PDGFA, SFRP1, and CSRP2 (Figure 5c). Our assessment of the nomogram for HRGs indicates that HRGs have high prognostic power (Figure 5d). According to the results of the survival analysis, patients belonging to the high expression HRG group have much lower prognoses in the TCGA-HNSCC dataset and the validation set GSE65858 (*p* < 0.05) (Figure 5e,f). The prognostic DCA curve (Figure 5g) also shows that HRGs exert optimal prognostic efficacy at 1, 2, and 3 years. The prognostic restricted cubic spline suggests that the risk of prognosis increases significantly with the increase of HRGs (Figure 5h), while the analysis of ROC at 1 year, 2 years, and 3 years further verifies the excellent prognostic efficacy of HRGs (Figure 5i). We then performed multivariate Cox analysis based on the expression levels of model genes that were associated with HRGs to calculate the contribution of model genes. The result that was shown (Figure 5j) was that TRMT112 had the highest contribution of genes.

### 4.5. Spatial Transcriptomic Localization Analysis

**Figure 6:**
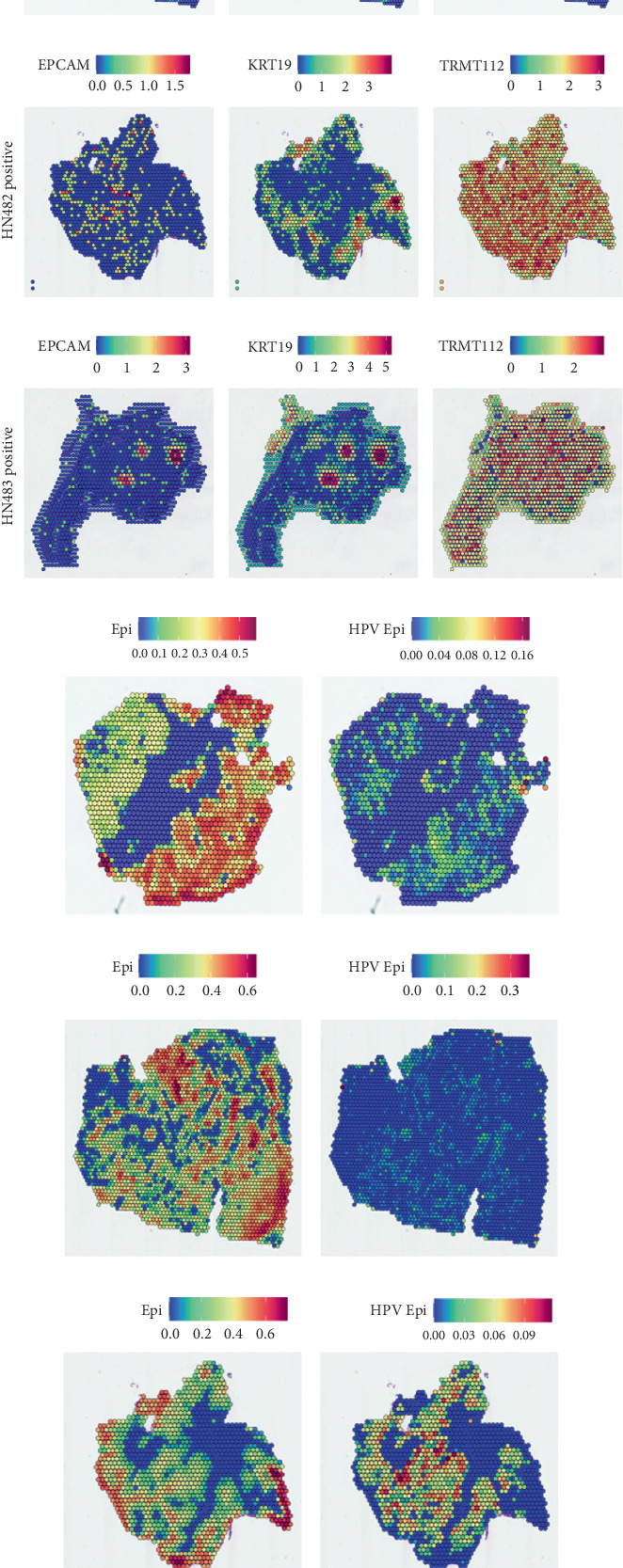
Spatial transcriptomics localization analysis. (a) The enrichment levels of TRMT112, EPCAM, and KRT19 in HN481 negative. (b) The enrichment levels of TRMT112, EPCAM, and KRT19 in HN487 negative. (c) The enrichment levels of TRMT112, EPCAM, and KRT19 in HN482 positive. (d) The enrichment levels of TRMT112, EPCAM, and KRT19 in HN483 positive. (e) The expression differences between HPV+ and HPV− epithelium in HN481 negative. (f) The expression differences between HPV+ and HPV− epithelium in HN487 negative. (g) The expression differences between HPV+ and HPV− epithelium in HN482 positive. (h) The expression differences between HPV+ and HPV− epithelium in HN483 positive.

**Figure 7:**
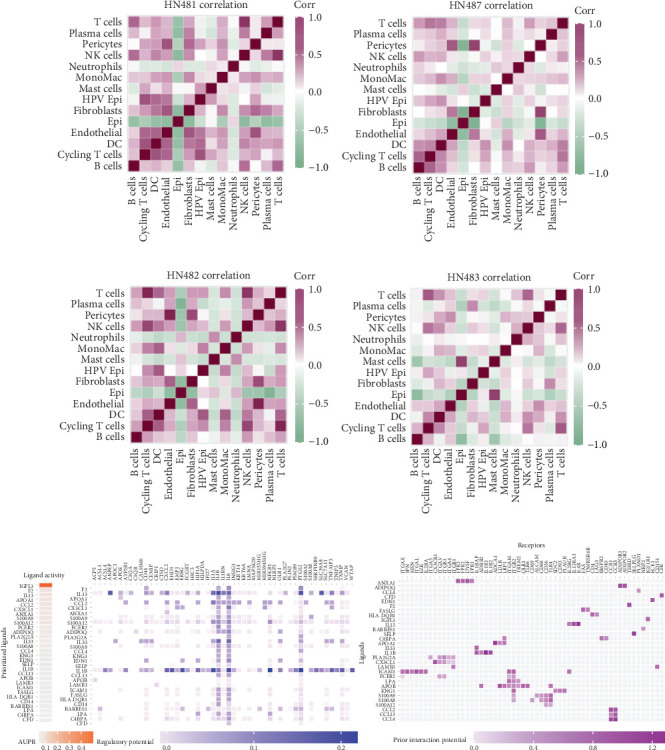
Spatial colocalization and NichNet cell communication. (a) Spatial cell colocalization in HN481 negative. (b) Spatial cell colocalization in HN487 negative. (c) Spatial cell colocalization in HN482 positive. (d) Spatial cell colocalization in HN483 positive. (e) NichNet differential cell communication reveals differences in communication between HPV+ epithelium and mononuclear macrophages in HPV+ and HPV− tissues.

We first conducted spatial localization using TRMT112 and the epithelial key marker genes EPCAM and KRT19. We observed significant expression of TRMT112 in malignant tumor epithelium and even higher expression in HPV+ epithelium (Figures 6a, 6b, 6c, and 6d). The HPV+ epithelium shows the presence of EPCAM and KRT19 expression at the same place, but exclusive to HPV− epithelium. To shed more light on the HPV epithelium expression in tumors, we performed spotlight deconvolution on markers. HPV+ epithelium is highly enriched in HPV+ tumors; it is sparsely distributed in HPV− tumors. HPV+ epithelium is generally localized in the center of the tumors (Figures 6e, 6f, 6g, and 6h). To further specify the spatial localization between HPV+ epithelium and other cells, we performed CARD for further analysis of spatial colocalization. The findings suggest that the HPV− epithelium displays a stronger relationship with different immune cell populations than HPV+ epithelium, which shows a much weaker relationship. Nonetheless, HPV+ epithelium was significantly spatially colocalized with mononuclear macrophages, with enrichment at higher levels in HPV+ tumors (Figures 7a, 7b, 7c, and 7d). In order to further validate this putative communication, we calculated the differential communication of HPV+ epithelium with mononuclear macrophages in HPV+ and HPV− tissues using the NicheNet package. Findings revealed that IL-13-, IL-1B-, and TGF-*β*-related receptor ligands showed significant enrichment between the two (Figure 7e).

### 4.6. Immune Infiltration Analysis

**Figure 8:**
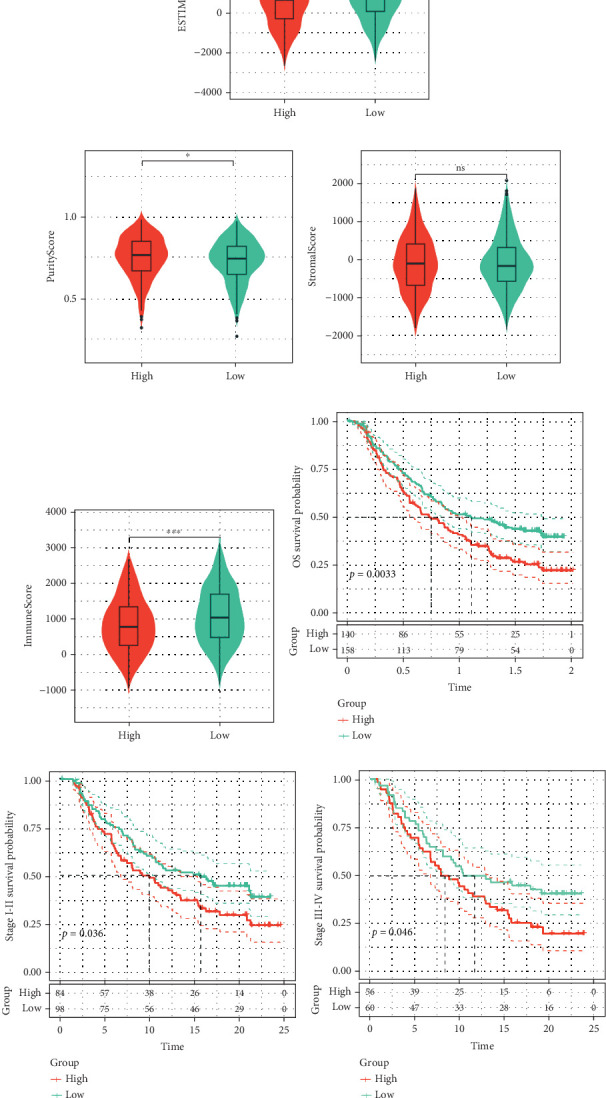
Immune infiltration analysis. (a) Immune infiltration analysis. (b–e) ESTIMATEScore, ImmuneScore, PurityScore, StromalScore, and ImmuneScore for high- and low-risk groups. (f) Prognosis (OS) of high- and low-risk groups in the IMvigor210 cohort. (g) Prognosis of high- and low-risk groups in Stages I–II of the IMvigor210 cohort. (h) Prognosis of high- and low-risk groups in Stages III–IV of the IMvigor210 cohort. (i, j) Proportion and differences in immunotherapy response between high- and low-risk groups. (k) Submap analysis. ⁣^∗^*p* value < 0.05, ⁣^∗∗^*p* value < 0.01, ⁣^∗∗∗^*p* value < 0.001, and ⁣^∗∗∗∗^*p* value < 0.0001; ns represents insignificant difference.

We performed an immune infiltration analysis utilizing CIBERSORT, EPIC, MCPcounter, and TIMER, focusing on high- and low-risk groups of HRGs. Our findings demonstrated that the levels of CD8 T cells, CD4 T cells, and B cells—key effector cells—were notably elevated in the low HRG group in comparison to the HRG group (Figure 8a). The subsequent ESTIMATE analysis indicated that the high-risk group exhibited a reduced tumor purity score, as well as lower estimate and immune scores (Figures 8b, 8c, 8d, and 8e). Additionally, deconvolution of the HRG model based on the IMvigor210 data cohort revealed that patients with high levels of HRGs had worse prognoses (Figure 8f). When we stratified the data by clinical stage, we discovered that patients in both Stages I–II and Stages III–IV experienced poorer prognoses (Figure 8g,h). Furthermore, it was noted that those in the high expression HRG group had an unfavorable response to atezolizumab, reflected by a significant rise in the PD/SD ratio (Figure 8i,j). In parallel, submap analysis suggested that the high expression HRG group exhibited a diminished response to immunotherapy (Figure 8k).

### 4.7. Single-Gene Immune Infiltration Analysis and Functional Enrichment

**Figure 9:**
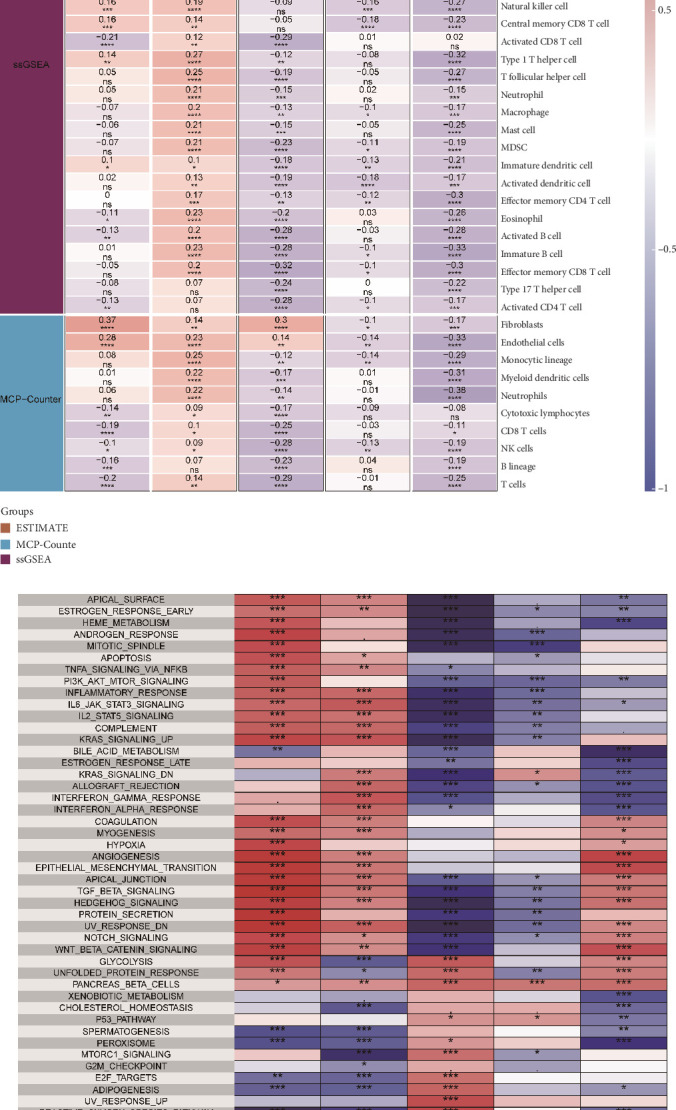
Single-gene immune infiltration and functional enrichment. (a) Single-gene immune infiltration analysis. (b) Single-gene functional enrichment analysis. ⁣^∗^*p* value < 0.05, ⁣^∗∗^*p* value < 0.01, ⁣^∗∗∗^*p* value < 0.001, and ⁣^∗∗∗∗^*p* value < 0.0001; ns represents insignificant difference.

The analysis of immune infiltration related to individual genes reveals that TRMT112 has a negative association with the majority of cytotoxic immune cells (*p* < 0.05) (Figure 9a). This observation may help explain our earlier analysis, which indicated that the high-risk cohort displayed a diminished effectiveness in immune responses. Moreover, the spatial communication assessments showed a reduced level of colocalization with mononuclear macrophages. We believe there is a valid basis for suggesting that TRMT112 could play a role in the reduced tumor immune infiltration seen in the high-risk group. Furthermore, the analysis focused on single-gene enrichment points to a positive correlation between TRMT112 and cellular immunity pathways, including E2F targets, MYC targets V1/2, and mTORC1 signaling (Figure 9b).

### 4.8. Single-Gene Prognosis and Pancancer Analysis

**Figure 10:**
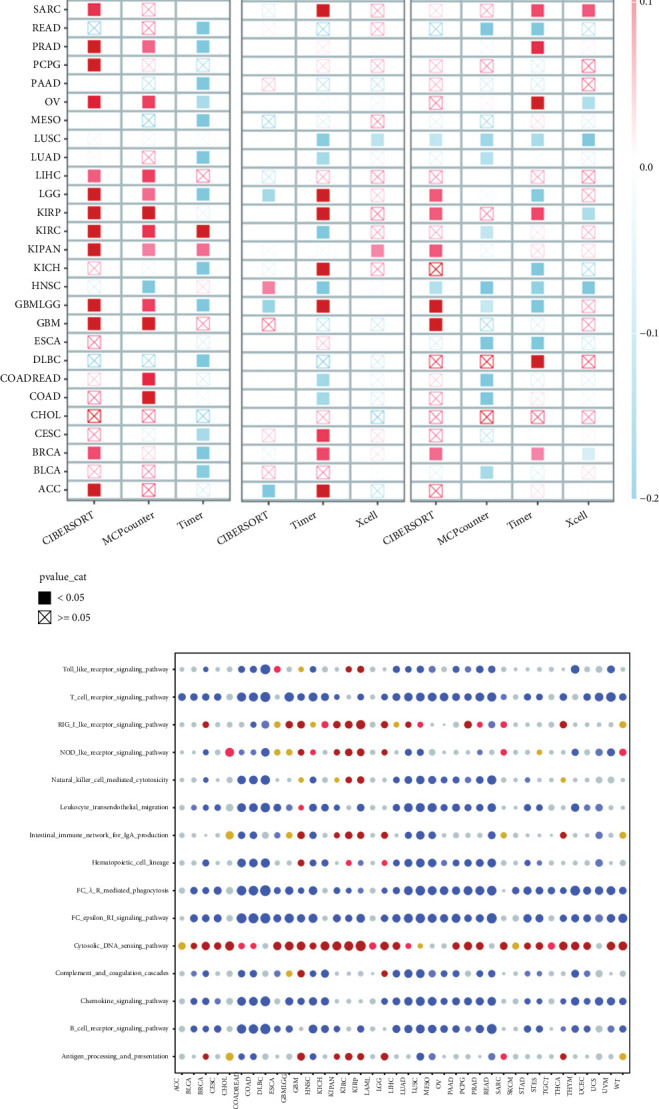
Prognosis of single genes and pancancer analysis. (a) Area infiltration analysis of single genes across pancancer. (b) Enrichment analysis of inflammatory pathway correlation related to single genes.

By integrating our preceding analysis of immune infiltration, we further assessed the relationship between TRMT112 and the populations of CD8, CD4, and B cells from a comprehensive cancer perspective. Our findings indicate that TRMT112 demonstrates considerable variability across various tumors, yet it generally displays a negative correlation in HNSCC (Figure 10a), thus reaffirming the conclusions of our earlier study. Additionally, an analysis of inflammatory pathway enrichment centered on TRMT112 suggests that it is inversely related to inflammatory responses in the majority of tumors. This points to the potential for TRMT112 to contribute to the establishment of a “cold tumor” within the TME. Consequently, this may explain the poorer outcomes seen in the high-risk group during immunotherapy (Figure 10b).

### 4.9. Experimental Validation

**Figure 11:**
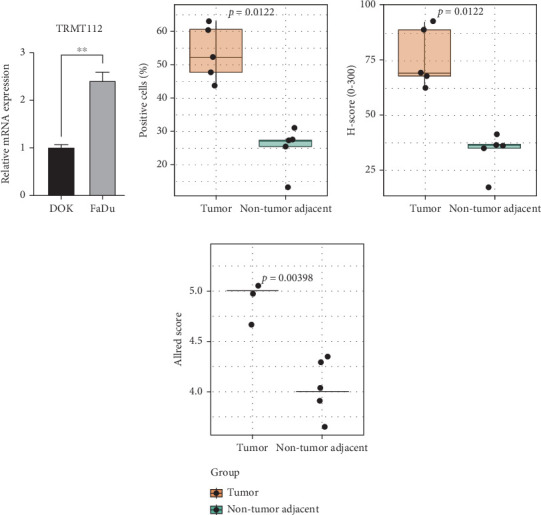
QPCR analysis and immunohistochemical analysis of TRMT112 expression in HNSCC. (a) Verify the differential expression of TRMT112 in DOK and FaDu cell lines through QPCR. (b–d) Boxplots show the percentage of positive cells, H-score, and Allred score. TRMT112 expression was significantly higher in tumor tissues compared with adjacent tissues (*p* = 0.012, *p* = 0.012, and *p* = 0.004, respectively; Wilcoxon rank-sum test). ⁣^∗∗^*p* value < 0.01.

In vitro experiments were conducted using DOK and FaDu cell lines, and the relative gene expression levels of TRMT112 were evaluated by QPCR. It was found that the expression level of TRMT112 in the FaDu group was significantly higher than that in the control group (*p* < 0.05, Figure 11a). Immunohistochemical staining demonstrated that TRMT112 protein expression was markedly elevated in tumor tissues compared with matched nontumor adjacent tissues (*n* = 5 per group). Quantitative image analysis showed that the proportion of positive cells was significantly higher in tumor samples (53.5% ± 8.3%) than in adjacent tissues (31.6% ± 8.5%, *p* = 0.012). Consistently, the H-score of TRMT112 immunoreactivity was greater in tumor tissues (76.2 ± 13.6) compared with nontumor adjacent tissues (45.5 ± 18.2, *p* = 0.012). In addition, the Allred score further supported stronger TRMT112 expression in tumor tissues (5.0 ± 0.0 vs.4.4 ± 0.9, *p* = 0.004). Collectively, these results indicate that TRMT112 is significantly upregulated in HNSCC relative to adjacent nontumor tissues (Figures 11b, 11c, and 11d).

## 5. Discussion

This research utilizes a range of techniques, such as single-cell sequencing, spatial transcriptomics, and machine learning, to thoroughly investigate the intricate molecular pathways and characteristics of the immune microenvironment in HPV-related HNSCC. By employing hdWGCNA and GeneNMF, we effectively pinpointed crucial cellular subpopulations in HPV-positive HNSCC and their interactions within the TME. This analysis demonstrates the impact of HPV infection on tumor biological behavior and immune evasion strategies through the modulation of particular cellular subsets. In a related study, Cillo et al. analyzed tumor-infiltrating leukocytes from 18 HPV-positive and 8 HPV-negative HNSCC patients using single-cell transcriptomic techniques. Their results indicated that the expression profiles of B cells and CD4+ T-cell subtypes in HPV-positive HNSCC differed significantly from those in HPV-negative cases, aligning with our observations. Patients with HPV-positive HNSCC tend to have a higher response rate to immunotherapy, which correlates with the pronounced expression of HPV-positive tumor cell subtypes in pathways associated with immune response [38]. These distinct cellular groups could facilitate the stimulation of immune checkpoints via intricate interactions with immune cells, consequently improving the effectiveness of immunotherapy. Previous studies have shown that cytotoxic T cells in HPV+ OPSCC (oropharyngeal squamous cell carcinoma) exhibit greater heterogeneity and have closer interactions with cancer cell clusters and other T-cell clusters, suggesting that HPV infection may enhance the immune response by influencing intercellular communication [14, 39]. To investigate the specific regulation of the immune microenvironment in HPV-positive HNSCC, we conducted an analysis of immune infiltration using single-cell, spatial transcriptomics, and conventional transcriptomic techniques. Our findings suggest that TRMT112 may serve as a potentially significant gene in this regulatory context. TRMT112 acts as a key enzyme mainly involved in m6A and m7G RNA methylation modifications. These modifications are crucial at multiple levels, including the regulation of gene expression, mRNA stability, and protein translation [40]. In HNSCC, the elevated levels of TRMT112 might facilitate the m6A modification of genes associated with tumors, which can improve their stability and translation effectiveness, ultimately leading to increased proliferation, invasion, and metastasis of cancer cells. Recent studies have shed light on the role of TRMT112 in HNSCC and other cancers. For instance, a pancancer analysis has revealed that TRMT112 expression is positively correlated with SART1 expression in various cancers, including HNSCC [41]. SART1 is a bicistronic gene that plays a crucial role in the initiation and development of HNSCC. This suggests that TRMT112 might promote tumorigenesis and development by interacting with SART1. Additionally, TRMT112 has been implicated in modulating RNA metabolism and transport pathways, which are essential for cancer progression.

Moreover, TRMT112 has been shown to be involved in the regulation of noncoding RNAs, such as lncRNA, miRNA, and tRNA, which participate in various biological processes, including cell proliferation, apoptosis, metastasis, and differentiation. These findings highlight the potential of TRMT112 as a prognostic predictor and an immunomodulatory factor in cancer. The m7G modification mediated by TRMT112 may also influence the expression of cell cycle regulatory genes, further promoting the malignant progression of tumor cells [42, 43]. It is noteworthy that TRMT112 plays a crucial role in the m7G methylation process, which has been confirmed to promote the transcription of ATF5, thereby driving tumor occurrence and progression [44]. This mechanism has been noted across different tumors, indicating that TRMT112 could be crucial in its oncogenic impacts by modulating ATF5 expression via m7G methylation. The elevated levels of TRMT112 in HNSCC might influence the expression of genes linked to immune suppression through m6A modification [45], potentially driving a transition in the TME toward an immunosuppressive phenotype [46]. The condition referred to as “cold tumor” allows tumor cells to more successfully escape detection and attacks from the immune system, which clarifies the observation that patients exhibiting high levels of TRMT112 expression tend to have less favorable outcomes with immunotherapy. Despite this, immunotherapy remains a promising avenue for HPV+ HNSCC treatment. Research indicates that patients with HPV+ HNSCC frequently experience improved survival rates when receiving anti-PD-1/PD-L1 antibody therapies. For example, one study revealed that HPV+ patients with PD-L1 positivity showed the greatest overall survival (OS) benefit when treated with nivolumab. Clinical trials such as KEYNOTE-040 and KEYNOTE-048 have also confirmed the efficacy of pembrolizumab in patients with HPV+ HNSCC [47]. Nonetheless, immunotherapy continues to encounter several obstacles in the context of HPV+ HNSCC. Despite the overall positive response to immunotherapy among patients with HPV+ HNSCC, there exists a group of individuals who do not show any response to the treatment. This lack of responsiveness may be linked to immunosuppressive mechanisms present within the TME. Additionally, the m7G modification mediated by TRMT112 could facilitate tumor development and progression through the upregulation of ATF5 transcription, thereby complicating the immunotherapy landscape. In order to address these challenges, researchers are investigating a variety of new strategies for immunotherapy. For example, current clinical trials are assessing the efficacy of combining anti-PD-1/PD-L1 inhibitors with other forms of immunomodulators, such as anti-CTLA-4 inhibitors. Additionally, HPV-specific immunotherapy strategies, such as HPV-specific therapeutic vaccines and HPV-specific adoptive T-cell therapy, have also shown potential in research [48].

In addition to the promising efficacy of the prognostic model demonstrated in our study, it is crucial to highlight its clinical significance and applicability across different patient subgroups. Our findings indicate that the model not only provides robust prognostic biomarkers for HPV-positive HNSCC but also exhibits good generalizability in predicting the prognosis of patients at various clinical stages, including early and late stages. This is further supported by its accurate prediction of immunotherapy response in the IMvigor210 cohort, even when stratified by different stages. This suggests that the model has the potential to serve as a valuable clinical tool for guiding treatment decisions and identifying patients who may benefit most from specific therapies. Given the increasing incidence of HPV-associated HNSCC and the need for more precise prognostic and therapeutic strategies, our model offers a significant advancement in the field, providing actionable targets for precision immunotherapy and contributing to improved patient outcomes.

Despite some advancements made in understanding the molecular mechanisms and immune microenvironment traits of HPV+ HNSCC, this study has notable limitations. To begin with, our investigation relies predominantly on single-cell sequencing and bulk transcriptomic data sourced from public repositories, which means that direct analysis of samples from primary HPV+ HNSCC patients is absent. This gap could hinder our nuanced comprehension of specific cell subpopulations and their associated molecular mechanisms. To enhance the biological insights of the key cell subpopulations and genes identified, future research should include a greater number of primary samples for validation. Additionally, our inquiry is mainly centered on the immune therapy response mechanisms in HPV+ HNSCC, whereas the immune therapy approaches for HPV− HNSCC remain relatively unexplored. Further studies could delve into the immune microenvironment features of HPV− HNSCC, aiming to discover new immunotherapeutic targets and improve treatment effectiveness for patients with HPV− HNSCC. Lastly, as single-cell multiomic technologies, such as single-cell proteomics and metabolomics, continue to evolve, impending research could merge these various omic strategies to thoroughly examine the molecular properties and biological processes of HPV+ HNSCC from diverse angles, thereby establishing a more robust theoretical framework for crafting targeted treatment strategies.

## 6. Conclusion

In conclusion, we developed a prognostic model that is intricately linked to HPV+ HNSCC by utilizing bulk transcriptomics, spatial transcriptomics, and single-cell data. Our research delved into the intricate interactions among critical HNSCC subpopulations and immune cells through multiomic approaches. This investigation uncovered significant factors that may affect the limited effectiveness of immunotherapy in HPV+ HNSCC. Furthermore, by employing a comprehensive screening strategy, we identified important prognostic genes that offer fresh perspectives for establishing treatment targets associated with HNSCC.

## Data Availability

The data that support the findings of this study are available from the corresponding authors upon reasonable request.

## Supporting Information

Supporting Information Table S1: Additional supporting information can be found online in the Supporting Information section. The performance of 101 predictive models in training and testing cohorts.
