# Supplementary material for: Multiomic Landscape Uncovers TRMT112 as a Central Driver of HPV-Positive Head and Neck Squamous Cell Carcinoma
Source: Hum Mutat. 2025 Nov 4;2025:5308441. doi: 10.1155/humu/5308441 (PMC12605863; doi:10.1155/humu/5308441)
Supplement: Supporting Information Table S1: — Additional supporting information can be found online in the Supporting Information section. The performance of 101 predictive models in training and testing cohorts. [file 5308441.f1.docx]

| **The performance of 101 predictive models in training and testing cohorts.** | | | | | | | |
| --- | --- | --- | --- | --- | --- | --- | --- |
| **Model** | **TCGA-CRC** | **GSE17536** | **GSE17537** | **GSE29621** | **GSE38832** | **GSE39582** | **GSE72970** |
| Lasso + StepCox [both] | 0.749 | 0.684 | 0.723 | 0.702 | 0.726 | 0.678 | 0.664 |
| survival-SVM | 0.708 | 0.651 | 0.751 | 0.658 | 0.693 | 0.621 | 0.577 |
| CoxBoost + survival-SVM | 0.727 | 0.655 | 0.761 | 0.652 | 0.699 | 0.597 | 0.554 |
| Ridge | 0.737 | 0.641 | 0.742 | 0.656 | 0.68 | 0.6 | 0.576 |
| Lasso + survival-SVM | 0.726 | 0.643 | 0.743 | 0.646 | 0.685 | 0.603 | 0.561 |
| SuperPC | 0.657 | 0.645 | 0.717 | 0.65 | 0.663 | 0.626 | 0.569 |
| CoxBoost + Ridge | 0.748 | 0.645 | 0.733 | 0.652 | 0.676 | 0.578 | 0.568 |
| Enet [alpha=0.1] | 0.752 | 0.642 | 0.726 | 0.651 | 0.66 | 0.576 | 0.579 |
| CoxBoost + Enet [alpha=0.1] | 0.751 | 0.644 | 0.731 | 0.648 | 0.659 | 0.568 | 0.569 |
| Enet [alpha=0.2] | 0.753 | 0.642 | 0.726 | 0.646 | 0.657 | 0.568 | 0.576 |
| Enet [alpha=0.3] | 0.753 | 0.643 | 0.731 | 0.651 | 0.647 | 0.564 | 0.578 |
| CoxBoost + Enet [alpha=0.3] | 0.752 | 0.643 | 0.735 | 0.653 | 0.645 | 0.561 | 0.573 |
| CoxBoost + Enet [alpha=0.2] | 0.752 | 0.643 | 0.733 | 0.648 | 0.651 | 0.563 | 0.569 |
| Enet [alpha=0.4] | 0.753 | 0.642 | 0.73 | 0.654 | 0.641 | 0.562 | 0.578 |
| CoxBoost + Enet [alpha=0.4] | 0.752 | 0.643 | 0.732 | 0.655 | 0.643 | 0.559 | 0.573 |
| Lasso + CoxBoost | 0.754 | 0.647 | 0.723 | 0.654 | 0.64 | 0.558 | 0.582 |
| Enet [alpha=0.5] | 0.753 | 0.643 | 0.731 | 0.652 | 0.638 | 0.561 | 0.578 |
| CoxBoost | 0.753 | 0.648 | 0.719 | 0.652 | 0.642 | 0.563 | 0.574 |
| CoxBoost + Enet [alpha=0.5] | 0.753 | 0.642 | 0.728 | 0.656 | 0.641 | 0.558 | 0.572 |
| Enet [alpha=0.6] | 0.753 | 0.642 | 0.727 | 0.652 | 0.637 | 0.56 | 0.577 |
| CoxBoost + Enet [alpha=0.6] | 0.753 | 0.643 | 0.725 | 0.657 | 0.638 | 0.556 | 0.572 |
| CoxBoost + Enet [alpha=0.7] | 0.753 | 0.643 | 0.725 | 0.656 | 0.638 | 0.556 | 0.573 |
| CoxBoost + Enet [alpha=0.8] | 0.753 | 0.644 | 0.725 | 0.656 | 0.636 | 0.556 | 0.573 |
| Enet [alpha=0.8] | 0.754 | 0.642 | 0.726 | 0.654 | 0.633 | 0.558 | 0.576 |
| Enet [alpha=0.9] | 0.754 | 0.643 | 0.726 | 0.654 | 0.633 | 0.557 | 0.577 |
| Lasso | 0.754 | 0.643 | 0.727 | 0.654 | 0.633 | 0.556 | 0.576 |
| Enet [alpha=0.7] | 0.754 | 0.642 | 0.726 | 0.652 | 0.634 | 0.558 | 0.576 |
| CoxBoost + Enet [alpha=0.9] | 0.753 | 0.644 | 0.725 | 0.656 | 0.635 | 0.555 | 0.573 |
| CoxBoost + Lasso | 0.753 | 0.644 | 0.725 | 0.655 | 0.635 | 0.555 | 0.573 |
| Lasso + plsRcox | 0.752 | 0.64 | 0.722 | 0.656 | 0.639 | 0.556 | 0.569 |
| CoxBoost + plsRcox | 0.751 | 0.641 | 0.724 | 0.656 | 0.636 | 0.554 | 0.569 |
| CoxBoost + StepCox [forward] | 0.752 | 0.641 | 0.725 | 0.653 | 0.636 | 0.552 | 0.568 |
| Lasso + StepCox [forward] | 0.752 | 0.641 | 0.72 | 0.656 | 0.634 | 0.552 | 0.569 |
| RSF + survival-SVM | 0.717 | 0.616 | 0.725 | 0.626 | 0.675 | 0.589 | 0.532 |
| CoxBoost + SuperPC | 0.636 | 0.623 | 0.745 | 0.613 | 0.678 | 0.585 | 0.515 |
| StepCox [forward] | 0.751 | 0.639 | 0.709 | 0.632 | 0.625 | 0.554 | 0.577 |
| plsRcox | 0.751 | 0.639 | 0.709 | 0.631 | 0.625 | 0.554 | 0.577 |
| RSF + Ridge | 0.736 | 0.619 | 0.715 | 0.634 | 0.653 | 0.572 | 0.538 |
| RSF + Enet [alpha=0.1] | 0.737 | 0.618 | 0.716 | 0.639 | 0.646 | 0.565 | 0.542 |
| Lasso + SuperPC | 0.651 | 0.607 | 0.726 | 0.601 | 0.672 | 0.59 | 0.525 |
| RSF + plsRcox | 0.737 | 0.625 | 0.715 | 0.641 | 0.641 | 0.56 | 0.538 |
| RSF + StepCox [forward] | 0.737 | 0.625 | 0.714 | 0.64 | 0.641 | 0.559 | 0.54 |
| RSF + Enet [alpha=0.2] | 0.737 | 0.619 | 0.713 | 0.636 | 0.643 | 0.563 | 0.545 |
| RSF + Enet [alpha=0.3] | 0.737 | 0.62 | 0.711 | 0.635 | 0.641 | 0.561 | 0.545 |
| RSF + Enet [alpha=0.6] | 0.737 | 0.622 | 0.711 | 0.635 | 0.638 | 0.559 | 0.547 |
| RSF + Lasso | 0.737 | 0.623 | 0.713 | 0.636 | 0.635 | 0.558 | 0.548 |
| RSF + Enet [alpha=0.7] | 0.737 | 0.623 | 0.709 | 0.636 | 0.637 | 0.559 | 0.548 |
| RSF + Enet [alpha=0.5] | 0.737 | 0.621 | 0.713 | 0.633 | 0.637 | 0.559 | 0.547 |
| RSF + CoxBoost | 0.737 | 0.623 | 0.709 | 0.637 | 0.636 | 0.558 | 0.547 |
| RSF + Enet [alpha=0.9] | 0.737 | 0.623 | 0.711 | 0.634 | 0.636 | 0.558 | 0.548 |
| RSF + Enet [alpha=0.4] | 0.737 | 0.62 | 0.711 | 0.634 | 0.638 | 0.56 | 0.546 |
| RSF + Enet [alpha=0.8] | 0.737 | 0.623 | 0.709 | 0.635 | 0.636 | 0.558 | 0.547 |
| RSF + StepCox [both] | 0.733 | 0.633 | 0.683 | 0.654 | 0.612 | 0.548 | 0.554 |
| RSF + StepCox [backward] | 0.733 | 0.633 | 0.683 | 0.654 | 0.612 | 0.548 | 0.554 |
| StepCox [both] + Ridge | 0.751 | 0.628 | 0.692 | 0.636 | 0.61 | 0.549 | 0.563 |
| StepCox [backward] + Ridge | 0.751 | 0.628 | 0.692 | 0.636 | 0.61 | 0.549 | 0.563 |
| StepCox [both] + plsRcox | 0.749 | 0.631 | 0.691 | 0.632 | 0.607 | 0.544 | 0.572 |
| StepCox [backward] + plsRcox | 0.749 | 0.631 | 0.691 | 0.632 | 0.607 | 0.544 | 0.572 |
| StepCox [both] + Enet [alpha=0.9] | 0.75 | 0.629 | 0.686 | 0.637 | 0.605 | 0.546 | 0.572 |
| StepCox [backward] + Enet [alpha=0.9] | 0.75 | 0.629 | 0.686 | 0.637 | 0.605 | 0.546 | 0.572 |
| StepCox [both] + Enet [alpha=0.1] | 0.751 | 0.628 | 0.688 | 0.638 | 0.608 | 0.547 | 0.566 |
| StepCox [backward] + Enet [alpha=0.1] | 0.751 | 0.628 | 0.688 | 0.638 | 0.608 | 0.547 | 0.566 |
| StepCox [both] + Enet [alpha=0.8] | 0.75 | 0.629 | 0.686 | 0.637 | 0.605 | 0.546 | 0.572 |
| StepCox [backward] + Enet [alpha=0.8] | 0.75 | 0.629 | 0.686 | 0.637 | 0.605 | 0.546 | 0.572 |
| StepCox [both] + Enet [alpha=0.2] | 0.751 | 0.629 | 0.689 | 0.636 | 0.607 | 0.546 | 0.568 |
| StepCox [backward] + Enet [alpha=0.2] | 0.751 | 0.629 | 0.689 | 0.636 | 0.607 | 0.546 | 0.568 |
| StepCox [both] + Lasso | 0.75 | 0.629 | 0.686 | 0.637 | 0.605 | 0.545 | 0.572 |
| StepCox [backward] + Lasso | 0.75 | 0.629 | 0.686 | 0.637 | 0.605 | 0.545 | 0.572 |
| StepCox [both] + Enet [alpha=0.6] | 0.75 | 0.629 | 0.686 | 0.636 | 0.606 | 0.546 | 0.571 |
| StepCox [backward] + Enet [alpha=0.6] | 0.75 | 0.629 | 0.686 | 0.636 | 0.606 | 0.546 | 0.571 |
| CoxBoost + GBM | 0.898 | 0.63 | 0.686 | 0.599 | 0.66 | 0.561 | 0.538 |
| StepCox [both] + Enet [alpha=0.7] | 0.75 | 0.63 | 0.686 | 0.636 | 0.605 | 0.546 | 0.571 |
| StepCox [backward] + Enet [alpha=0.7] | 0.75 | 0.63 | 0.686 | 0.636 | 0.605 | 0.546 | 0.571 |
| Lasso + StepCox [backward] | 0.749 | 0.63 | 0.683 | 0.635 | 0.607 | 0.545 | 0.573 |
| StepCox [both] | 0.749 | 0.63 | 0.683 | 0.635 | 0.607 | 0.545 | 0.573 |
| StepCox [backward] | 0.749 | 0.63 | 0.683 | 0.635 | 0.607 | 0.545 | 0.573 |
| CoxBoost + StepCox [both] | 0.749 | 0.63 | 0.683 | 0.635 | 0.607 | 0.545 | 0.573 |
| CoxBoost + StepCox [backward] | 0.749 | 0.63 | 0.683 | 0.635 | 0.607 | 0.545 | 0.573 |
| StepCox [both] + Enet [alpha=0.4] | 0.75 | 0.629 | 0.686 | 0.635 | 0.606 | 0.546 | 0.57 |
| StepCox [backward] + Enet [alpha=0.4] | 0.75 | 0.629 | 0.686 | 0.635 | 0.606 | 0.546 | 0.57 |
| StepCox [both] + Enet [alpha=0.3] | 0.751 | 0.63 | 0.688 | 0.633 | 0.606 | 0.546 | 0.569 |
| StepCox [backward] + Enet [alpha=0.3] | 0.751 | 0.63 | 0.688 | 0.633 | 0.606 | 0.546 | 0.569 |
| StepCox [both] + CoxBoost | 0.751 | 0.631 | 0.686 | 0.635 | 0.604 | 0.544 | 0.572 |
| StepCox [backward] + CoxBoost | 0.751 | 0.631 | 0.686 | 0.635 | 0.604 | 0.544 | 0.572 |
| StepCox [both] + Enet [alpha=0.5] | 0.75 | 0.629 | 0.686 | 0.634 | 0.605 | 0.546 | 0.57 |
| StepCox [backward] + Enet [alpha=0.5] | 0.75 | 0.629 | 0.686 | 0.634 | 0.605 | 0.546 | 0.57 |
| CoxBoost + RSF | 0.969 | 0.609 | 0.673 | 0.614 | 0.676 | 0.554 | 0.543 |
| RSF + SuperPC | 0.642 | 0.607 | 0.68 | 0.619 | 0.663 | 0.583 | 0.508 |
| RSF | 0.971 | 0.59 | 0.663 | 0.604 | 0.685 | 0.565 | 0.548 |
| Lasso + GBM | 0.896 | 0.625 | 0.682 | 0.588 | 0.655 | 0.566 | 0.539 |
| RSF + GBM | 0.879 | 0.629 | 0.686 | 0.611 | 0.646 | 0.557 | 0.522 |
| GBM | 0.899 | 0.619 | 0.661 | 0.607 | 0.648 | 0.568 | 0.54 |
| StepCox [both] + survival-SVM | 0.739 | 0.617 | 0.688 | 0.61 | 0.617 | 0.555 | 0.551 |
| StepCox [backward] + survival-SVM | 0.739 | 0.617 | 0.688 | 0.61 | 0.617 | 0.555 | 0.551 |
| Lasso + RSF | 0.97 | 0.59 | 0.663 | 0.594 | 0.68 | 0.556 | 0.546 |
| StepCox [both] + GBM | 0.881 | 0.618 | 0.625 | 0.585 | 0.602 | 0.535 | 0.541 |
| StepCox [backward] + GBM | 0.881 | 0.618 | 0.625 | 0.585 | 0.602 | 0.535 | 0.541 |
| StepCox [both] + RSF | 0.967 | 0.591 | 0.622 | 0.592 | 0.593 | 0.542 | 0.549 |
| StepCox [backward] + RSF | 0.967 | 0.591 | 0.622 | 0.592 | 0.593 | 0.542 | 0.549 |
| StepCox [both] + SuperPC | 0.521 | 0.537 | 0.576 | 0.531 | 0.551 | 0.519 | 0.506 |
| StepCox [backward] + SuperPC | 0.521 | 0.537 | 0.576 | 0.531 | 0.551 | 0.519 | 0.506 |
